# TRPV1-mediated Pharmacological Hypothermia Promotes Improved Functional Recovery Following Ischemic Stroke

**DOI:** 10.1038/s41598-017-17548-y

**Published:** 2017-12-15

**Authors:** Zhijuan Cao, Adithya Balasubramanian, Steen E. Pedersen, Jonathan Romero, Robia G. Pautler, Sean P. Marrelli

**Affiliations:** 1 Department of Molecular Physiology and Biophysics-Cardiovascular Sciences Track, Baylor College of Medicine, Houston, TX 77030 USA; 2 Department of Molecular Physiology and Biophysics, Baylor College of Medicine, Houston, TX 77030 USA; 3 Department of Anesthesiology, Baylor College of Medicine, Houston, TX 77030 USA; 4 Department of Neurology, McGovern Medical School at UTHealth, Houston, TX 77030 USA

## Abstract

Hypothermia shows promise for stroke neuroprotection, but current cooling strategies cause undesirable side effects that limit their clinical applications. Increasing efforts have focused on pharmacological hypothermia as a treatment option for stroke. Previously, we showed that activation of a thermoregulatory ion channel, transient receptor potential vanilloid 1 (TRPV1), by dihydrocapsaicin (DHC) produces reliable hypothermia. In this study, we investigate the effects of TRPV1-mediated hypothermia by DHC on long-term ischemic stroke injury and functional outcome. Hypothermia initiated at 3.5 hours after stroke significantly reduced primary cortical injury. Interestingly, hypothermia by DHC also significantly reduced secondary thalamic injury, as DHC-treated stroke mice exhibited 53% smaller thalamic lesion size. DHC-treated stroke mice further demonstrated decreased neuronal loss and astrogliosis in the thalamus and less thalamic fiber loss by diffusion tensor imaging (DTI). Importantly, a single 8 hour treatment of hypothermia by DHC after stroke provided long-term improvement in functional outcome, as DHC-treated mice exhibited improved behavioral recovery at one month post-stroke. These findings indicate that TRPV1-mediated hypothermia is effective in reducing both primary cortical injury and remote secondary thalamic injury, and a single treatment can produce persistent effects on functional recovery. These data highlight the therapeutic potential for TRPV1 agonism for stroke treatment.

## Introduction

Stroke is the fifth leading cause of death and the leading cause of long-term adult disability in the United States^1^. For patients with ischemic stroke, best outcome is generally achieved by quickly restoring cerebral blood flow (recanalization) through thrombolytic therapy (r-tPA)^2^, endovascular thrombectomy^3^, or spontaneous reperfusion^4^. Nevertheless, even with a timely recanalization, some patients still experience sub-optimal outcomes^2,5^. Mild hypothermia (32–34 °C) has been shown to provide positive outcome for experimental stroke^6,7^. However, there are significant challenges for applying hypothermia as a therapy for stroke patients, including variable effectiveness in lowering body temperature, shivering, and other clinical complications (hypotension, cardiac arrhythmia, pneumonia, *etc*.). Increasing efforts have been focused on improving or developing cooling methods for awake subjects that can overcome these challenges so that more stroke patients can benefit from its clinical applications.

Modulating the body temperature by pharmacological strategies is a promising alternative method to achieve targeted whole body cooling accurately and efficiently with reduced adverse effects. Previously we have demonstrated a novel pharmacological method that involves the activation of Transient receptor potential vanilloid 1 (TRPV1)^8,9^ to induce rapid and consistent hypothermia in conscious rodents. TRPV1 is a thermosensitive ion channel highly expressed in warm-sensing nerve fibers of the autonomic thermoregulatory system^10^. TRPV1 can be activated by heat (>43 °C) and protons, as well as endogenous and exogenous agonists such as capsaicin and dihydrocapsaicin (DHC)^10,11^. Systemic pharmacological TRPV1 activation (TRPV1 agonism) induces rapid and sustained hypothermia in rodents, cynomologus monkeys and young cattle^8,12^. Previously our group^9^ and Muzzi *et al*.^13^ have reported that TRPV1 agonist induced hypothermia provides significant neuroprotection within the first few days following ischemic stroke in mice. We demonstrated that the acute neuroprotection by DHC is mediated specifically through TRPV1 channels and the resulting induced hypothermia^9^. However, despite its neuroprotective effects in the acute phase after stroke, it is unclear whether TRPV1-mediated hypothermia has long-term benefit on ischemic injury and functional recovery. It must be noted that ischemic injury can continue to develop over weeks or months. Furthermore, in addition to the primary injury region, delayed secondary degenerative injury can develop over the ensuing days and weeks in regions either directly adjacent or even non-adjacent to the primary injury. For instance, secondary degenerative injury has been reported in the thalamus after the distal middle cerebral artery occlusion (dMCAO). This progressive secondary thalamic injury contributes to somatosensory dysfunction and can affect the final neurological behavioral outcome^14^. Therefore, the current study investigates the long-term effects of TRPV1-mediated pharmacological hypothermia on both primary and secondary post-stroke injury and functional outcome during one month after stroke.

## Results

### DHC-induced hypothermia significantly attenuates primary cortical infarct at one month post-stroke

**Figure 1:**
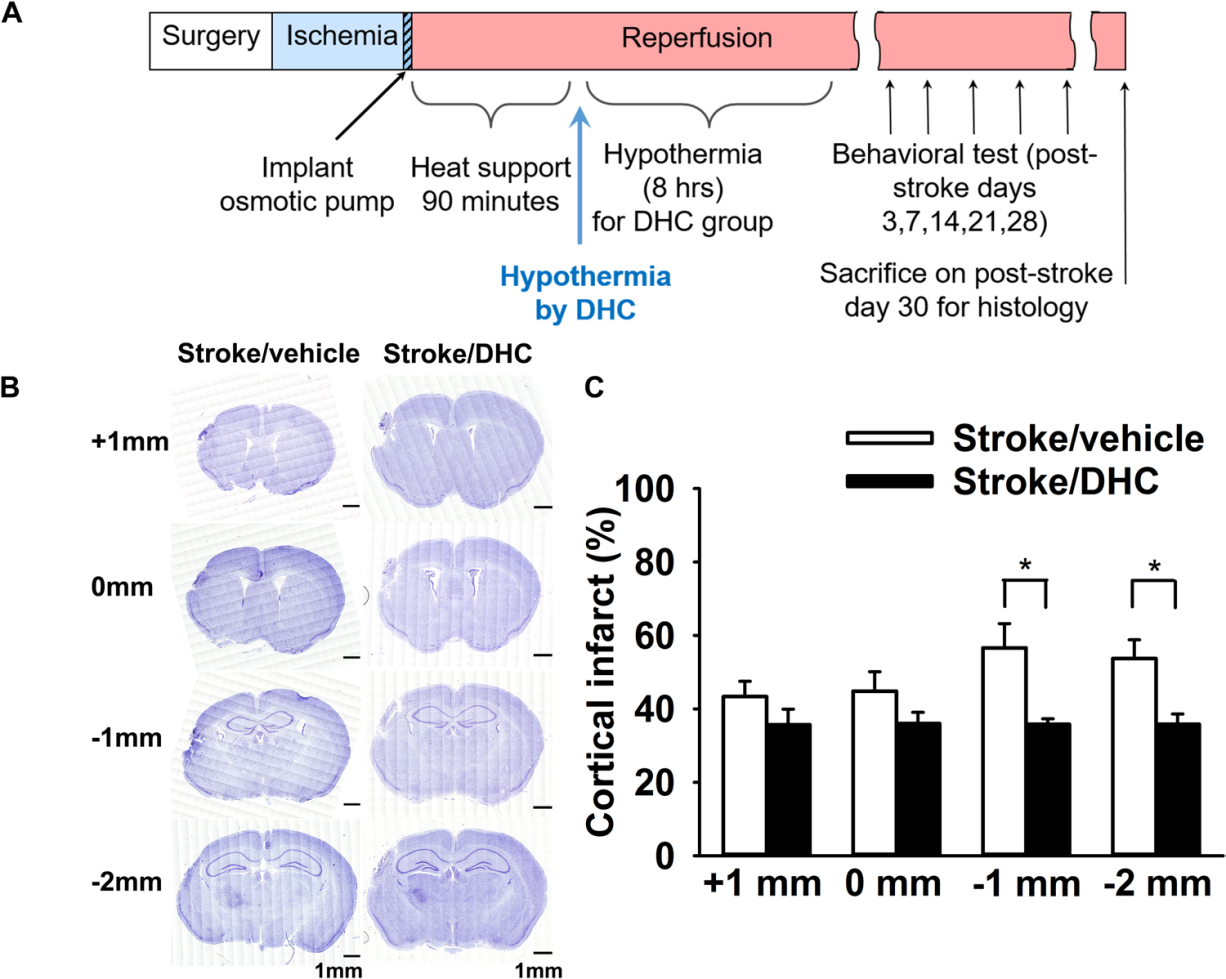
DHC-induced hypothermia significantly reduces cortical injury after one month post-stroke. (**A**) Schemes of experimental procedure. Ischemia was induced for 120 minutes followed by 90 minutes of reperfusion with heat support. Either DHC or vehicle was infused by an osmotic pump for 8 hours starting at 90-minute after reperfusion. Behavioral tests were performed on post-stroke day 3, 7, 14, 21 and 28. On post-stroke day 30, mice were sacrificed and brain samples were collected for further histology analysis (Nissl staining and immunostaining) in all groups. (**B**) Representative images of brain section at +1 mm, 0 mm, −1 mm and −2 mm from bregma stained by Nissl stain. Full section high resolution images were generated by stitching together a total of 400 confocal images (20× magnification). Scale bar = 1 mm. (**C**) Summary of cortical infarct area. Cortical infarct% = [(contralateral cerebral cortex area − ipsilateral cerebral cortex area + infarcted area)/contralateral cerebral cortex area] × 100. (t test, *p < 0.05, n = 10 in Stroke/vehicle and 8 in Stroke/DHC, data are expressed as mean ± SEM).

To investigate the long-term effects of DHC-induced hypothermia on cerebral ischemic stroke, a single treatment of DHC-induced hypothermia (8 hours duration) was initiated at 3.5 hours post-stroke. We then measured behavioral outcome through post-stroke day 28 and then harvested the brain for histological analyses on post-stroke day 30 (Fig. 1A). Coronal sections representing positions of −1 mm and −2 mm from bregma demonstrated significantly decreased infarct area in the Stroke/DHC group compared to the Stroke/vehicle group (Fig. 1B,C, *p < 0.05). At −1 mm from bregma, cortical infarct area decreased by 37% (35.8 ± 1.5% in Stroke/DHC vs. 56.6 ± 6.6% in Stroke/vehicle). At −2 mm from bregma, cortical infarct area decreased by 33% (35.8 ± 2.8% in Stroke/DHC vs. 53.7 ± 5.1% in Stroke/vehicle).

### DHC-induced hypothermia reduces secondary thalamic injury at one month post-stroke

**Figure 2:**
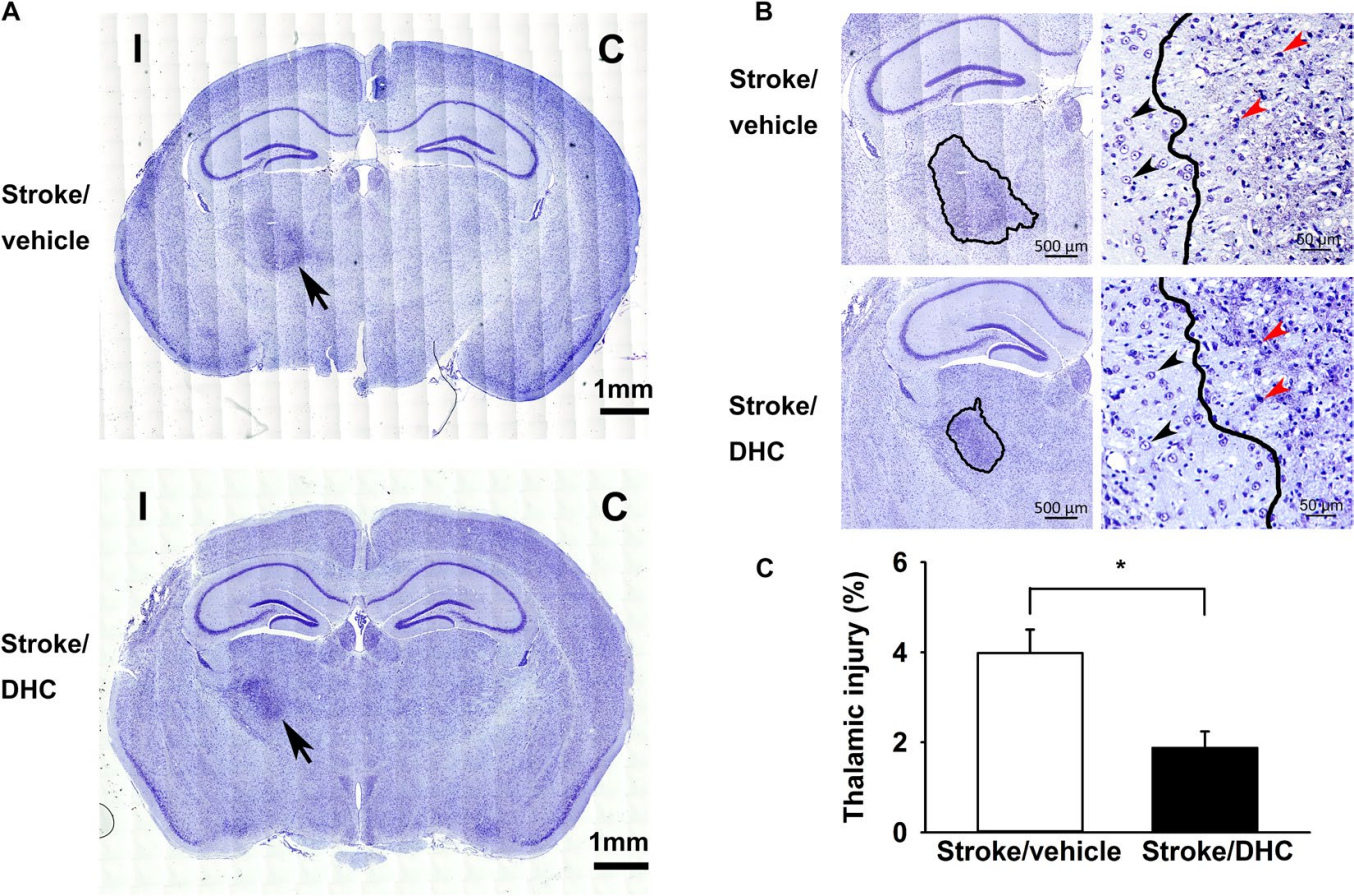
DHC-induced hypothermia significantly attenuates secondary thalamic injury at one month post-stroke. **(A)** Representative Nissl-stained images show a darkly stained region (arrow) in ipsilateral thalamus at one month post-stroke. (**C**) contralateral side, I: ipsilateral side. Scale bar = 1 mm. (**B**) Left: Enlarged images highlight the degenerative neuronal damage on the ipsilateral side (outlined by a solid line); scale bar = 500 µm. Right: Representative images from thalamic area at one month post-stroke from both groups show the well-defined boundary between degenerating and healthy neurons. Dense pyknotic-necrotic neuron bodies indicate the area of secondary thalamic neuron degeneration; scale bar = 50 µm. Black arrows indicate normal neurons and red arrows indicate typical neuronal debris. (**C**) Summary of ipsilateral thalamic injury. Thalamic injury% = thalamic injury area/total contralateral hemisphere area × 100. (t test, *p < 0.05, n = 10 in Stroke/vehicle and 8 in Stroke/DHC, data are expressed as mean ± SEM).

In Nissl-stained brain sections at one month post-stroke, we noticed a more darkly stained ipsilateral thalamic region in the sections taken at −2 mm from bregma (indicated by an arrow in Fig. 2A). We observed a well-defined area containing significant pyknotic stained debris and loss of normal neuronal structure (injury boundary indicated by a solid line in Fig. 2B; enlarged in Fig. 2B right panels). This type of secondary injury has been described as “thalamic neuronal degenerative injury” and has been reported following permanent middle cerebral artery occlusion and other types of focal cortical injury^15–17^. Both the Stroke/vehicle and Stroke/DHC groups had evidence of thalamic injury at one month post-stroke; however, secondary thalamic injury area was significantly reduced by 53% in the Stroke/DHC group (normalized injury: 1.9 ± 0.4% Stroke/DHC vs. 4.0 ± 0.5% Stroke/vehicle) (Fig. 2C, *p < 0.05).

**Figure 3:**
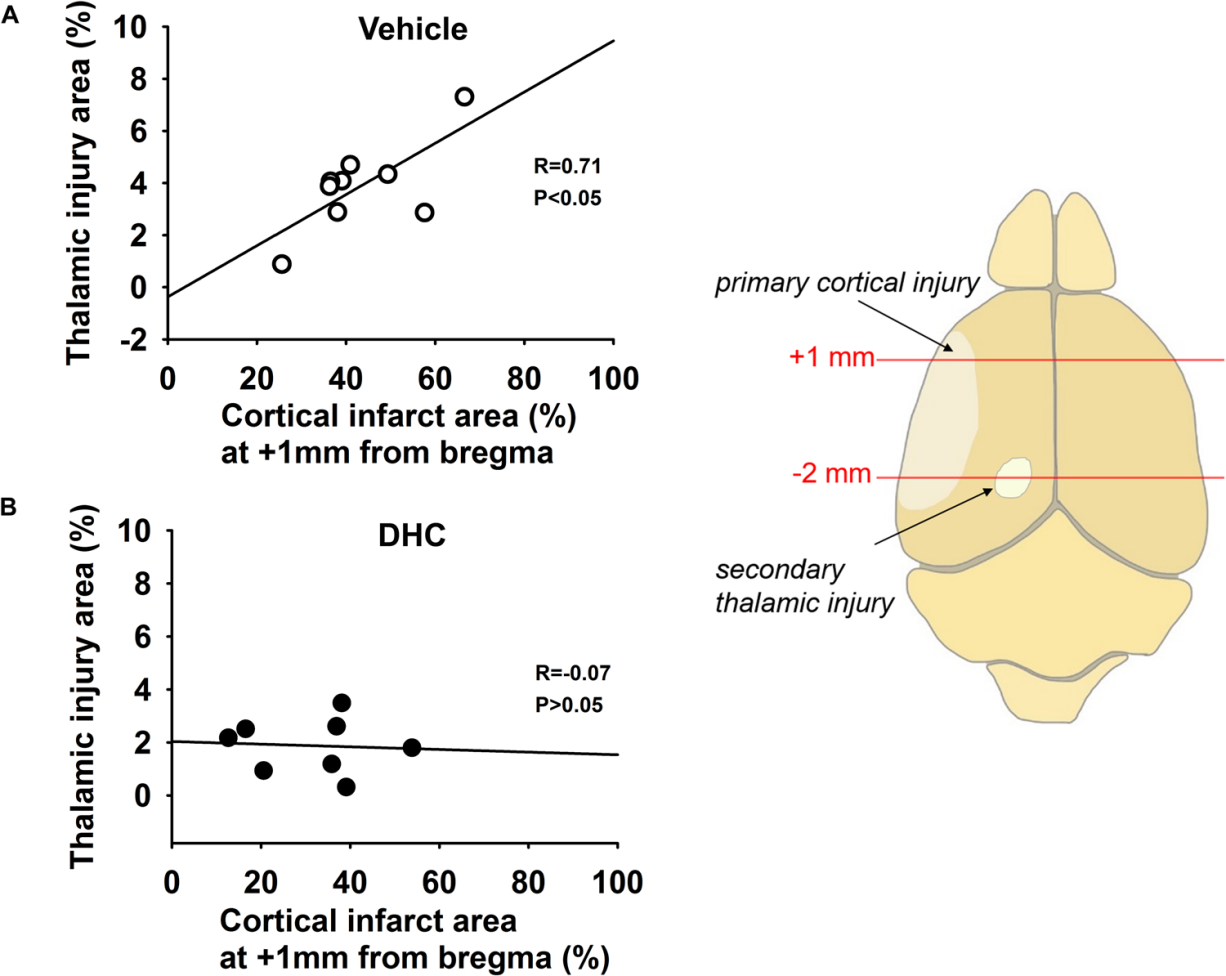
Correlation between primary cortical injury and secondary thalamic injury is disrupted in DHC-induced hypothermia treated mice at one month post-stroke. Thalamic injury area (from −2 mm section) plotted versus cortical infarct area (from +1 mm section). A significant correlation between the cortical injury and thalamic injury was found in the Stroke/vehicle group (**A**), but not in the Stroke/DHC group (**B**). Pearson correlation analysis and Parallel line analysis, R and P values are indicated within each plot. *p < 0.05, n = 9 in Stroke/vehicle and 8 in Stroke/DHC. Data are expressed as mean ± SEM). The diagram shows the section positions of cortical injury and thalamic injury.

We further performed correlation analysis to examine whether the reduced secondary thalamic injury with DHC-induced hypothermia was related to *A*) a decrease in primary cortical injury, or instead, *B*) a downstream attenuation of secondary injury. First, we analyzed which coronal section(s) demonstrated a positive correlation between cortical injury and thalamic injury in Stroke/vehicle mice. Regression analysis revealed that cortical infarct injury at +1 mm from bregma significantly and positively correlated with thalamic injury size (Fig. 3A, *P < 0.05). No significant correlation was evident at the other three positions (see Supplemental Fig. 2). Interestingly, regression analysis of Stroke/DHC brains demonstrated no correlation between thalamic and cortical injury from +1 mm sections (Fig. 3B) or any of the other sections. Furthermore, statistical comparison of regression line slopes demonstrated a significant difference between Stroke/vehicle and Stroke/DHC groups (*P < 0.05), supporting the possibility that DHC treatment results in an attenuation of secondary injury.

### DHC-induced hypothermia attenuates neuronal loss and reduces astrogliosis in the ipsilateral thalamus at one month post-stroke

**Figure 4:**
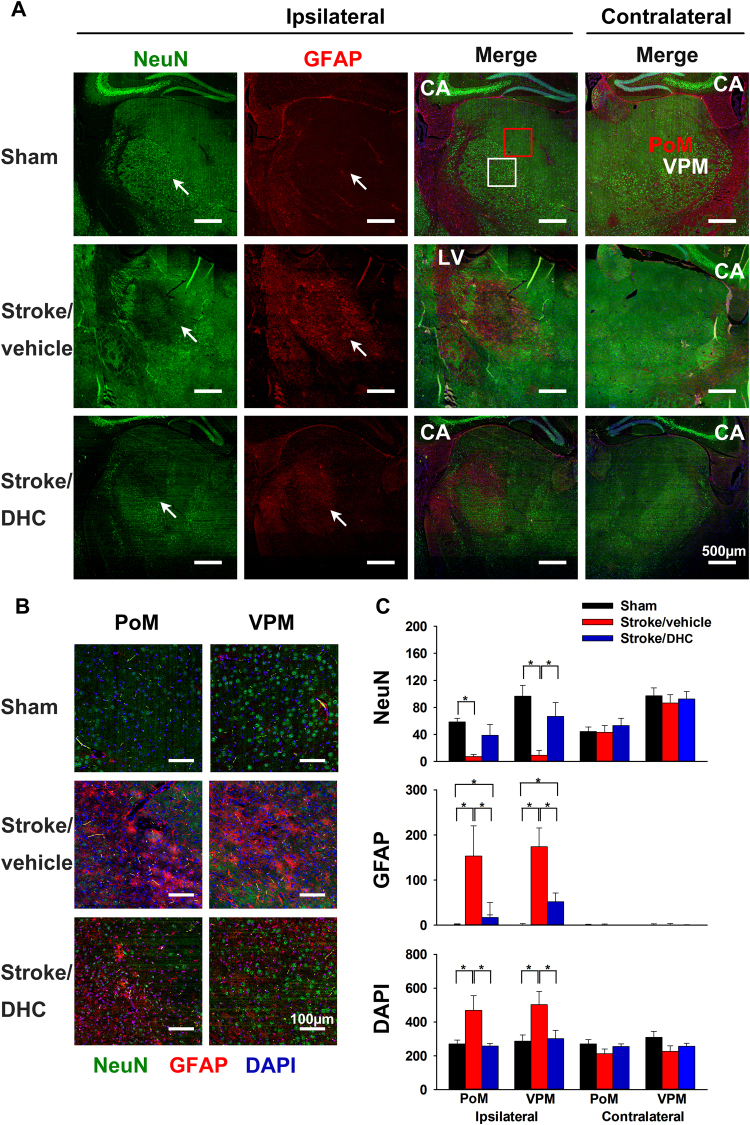
DHC-induced hypothermia attenuates neuron loss and reduces astrogliosis in the ipsilateral thalamus area after one month post-stroke. (**A**) Representative stitched immunofluorescence images show neuronal loss and reactive astrocytes clustered in the ipsilateral thalamic area at one month post-stroke. Arrows indicate the thalamus. Anti-NeuN stains normal neurons, anti-GFAP stains active astrocytes, DAPI stains nuclei. Scale bar = 500 µm. CA: cornu ammonis; LV: lateral ventricle. A red square marks the posteromedial complex (PoM) and a white square marks the ventral posteromedial nucleus (VPM) area on thalamus. (**B**) Representative enlarged images from PoM and VPM from ipsilateral sides. Scale bar = 100 µm. (**C**) Summary of cell numbers from PoM and VPM regions of the thalamus (Kruskal-Wallis one way ANVA and Mann-Whiney U test with Bonferronni correction for post hoc multiple comparisons, *p < 0.05, n = 6 in Sham, 4 in Stroke/vehicle and 5 in Stroke/DHC, data are expressed as median ± SEM).

Next, we examined whether DHC-mediated hypothermia affects neuronal loss and astrogliosis in the thalamus. Multiple 20x images were digitally stitched together to achieve a full high-resolution map of the thalamus at −2 mm from bregma. Co-labeling with NeuN and GFAP demonstrated considerable loss of neurons and abundant hypertrophic reactive astrocytes in the ipsilateral thalamus in the Stroke/vehicle group. Injury was most notable within the ventral posteromedial nucleus (VPM) and posteromedial complex (PoM) regions of the thalamus (Fig. 4A,B). In comparison, the Stroke/DHC group showed significantly less neuron loss, fewer reactive astrocytes, and lack of increased cellularity (Fig. 4C, *p < 0.05). No difference among the three groups was shown in the contralateral thalamus.

### DHC-induced hypothermia reduces thalamic fiber loss at one month post-stroke

**Figure 5:**
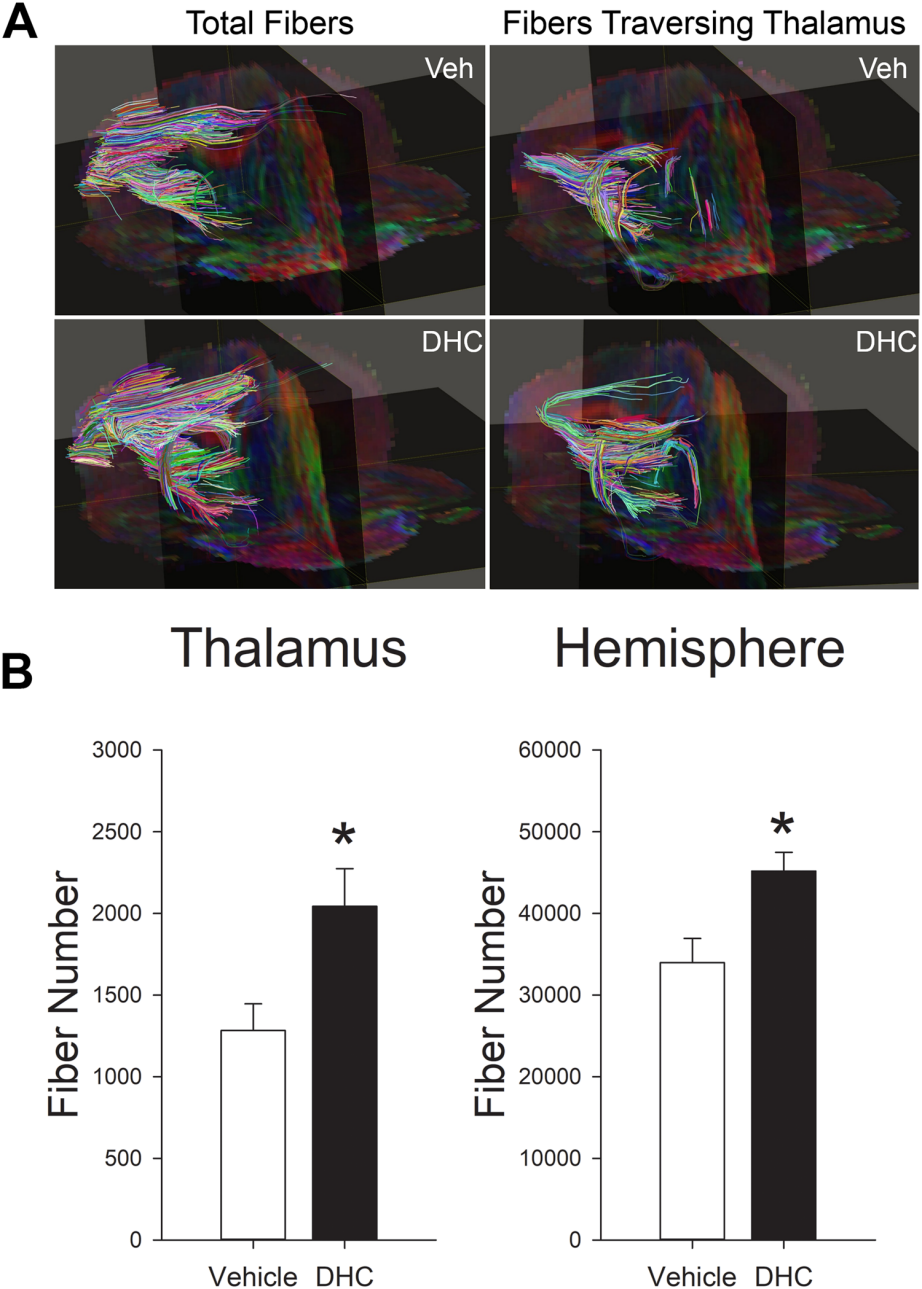
DHC-induced hypothermia protects against fiber loss in ipsilateral hemisphere and thalamus after one month post-stroke. **(A)** Representative diffusion tensor imaging (DTI) fiber tract map of stroke injury hemispheres from vehicle treated and DHC treated mice at one month post-stroke. Left side panels reflect total fibers from the ipsilateral hemisphere. Right side panels reflect only those fibers traversing the injury region of the thalamus. **(B)** Quantitative analysis of fiber density in ipsilateral thalamic regions and whole brain hemisphere between Stroke/vehicle and Stroke/DHC groups (t test, *P < 0.05, n = 4/group, data are expressed as mean ± SEM).

To further investigate the effects of DHC-induced hypothermia on secondary thalamic degenerative injury, we used diffusion tensor imaging (DTI) to evaluate cortical-thalamic fiber tract density in vehicle and DHC-induced hypothermia treated stroke mice at one month post-stroke. Fractional anisotropy (FA) images were used to map injury within the cortex and thalamus and register fiber tracts. DTI fiber tract analysis was used to evaluate fiber density, specifically within the thalamic regions (Fig. 5). Quantitative fiber tract analysis between groups demonstrated a significantly greater number of fiber tracts traversing the ipsilateral thalamus of the Stroke/DHC group versus the Stroke/vehicle group (2044 ± 230 vs. 1283 ± 163, Fig. 5B, *P < 0.05). The number of fiber tracts traversing the contralateral thalamus was not statistically different between groups (2329 ± 820 vs. 1884 ± 509). In addition, there was a significantly greater number of fibers by hemisphere in the Stroke/DHC group compared with Stroke/vehicle group (45166 ± 2302 vs. 33978 ± 2952, Fig. 5B, *P < 0.05).

### DHC-induced hypothermia improves long-term neurologic function following ischemic stroke

**Figure 6:**
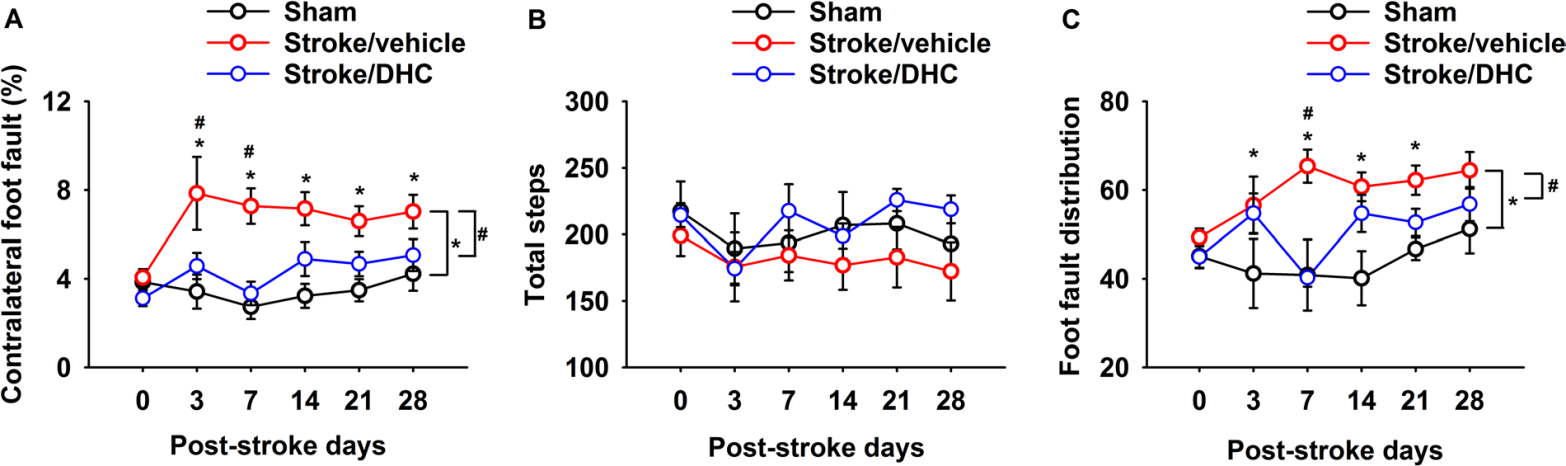
DHC-induced hypothermia significantly improves long-term neurologic function during one month post-stroke. (**A**) Summary of contralateral side foot faults as a percent of total steps. (**B**) Summary of total steps during the two-minute test period. (**C**) Summary of the foot fault distribution, presented as the percentage of contralateral side foot faults divided by total foot faults (two-way repeated ANOVA with Holm-Sidak test, *p < 0.05 Sham *vs*. Stroke/vehicle; ^#^p < 0.05 Stroke/vehicle *vs*. Stroke/DHC, n = 8 for Sham, n = 11 for Stroke/vehicle and n = 7 in Stroke/DHC, data are expressed as mean ± SEM).

No difference was evident in baseline foot fault values in all groups. However, following dMCAO, the Stroke/vehicle group showed a significantly elevated foot fault percentage compared to the Sham group (group difference). Individual comparisons demonstrated a significantly higher percentage of contralateral foot faults through 28 days (Fig. 6A, *p < 0.05). Stroke/DHC group mice achieved significantly improved performance compared with the Stroke/vehicle group, demonstrating significantly lower contralateral foot fault percentage on post-stroke day 3 and day 7 (Fig. 6A, ^#^p < 0.05). There was no significant difference in foot fault percentage between Sham and Stroke/DHC groups or with total steps taken among all three groups (Fig. 6B). Since cortical damage results in contralateral motor dysfunction, we also specifically examined the frequency of contralateral foot faults as a percentage of the total. The Stroke/vehicle group demonstrated a significantly greater frequency of foot fault on the contralateral side, whereas Sham and Stroke/DHC mice demonstrated a more equally-distributed frequency of foot fault (Fig. 6C, *p < 0.05, ^#^p < 0.05).

## Discussion

We demonstrate that TRPV1-mediated pharmacological hypothermia reduces both primary cortical infarct area and secondary thalamic injury area following cerebral ischemia/reperfusion. The TRPV1-mediated hypothermia also attenuates secondary neuronal loss and astrogliosis in the ipsilateral thalamus and reduces the thalamic fiber loss at one month post-stroke. Finally, a single eight-hour treatment of TRPV1-mediated hypothermia provides persistent behavioral recovery through one month post-stroke. To our knowledge, this is the first study to demonstrate that application of pharmacological hypothermia (or any form of hypothermia) can protect against remote secondary injury and provide lasting recovery of function following ischemic stroke.

Mild hypothermia has been demonstrated to provide brain protection for patients with witnessed cardiac arrest and for neonatal ischemia^18,19^. Application of induced hypothermia to adult stroke patients, however, brings significantly greater challenges^20^. Given the numerous problems with traditional surface cooling methods^21^, alternative methods of induced hypothermia have been explored for application in stroke^22^. Unfortunately, the current state of the art in cooling conscious stroke patients below 34 °C is still ineffective for many patients, and thus improved methods are still needed^23^. Ours and other studies have demonstrated that TRPV1-mediated pharmacological hypothermia decreases the cortical injury and provides neuroprotection after 24 hours cerebral reperfusion in the stroke model^9,13^. However, an acute protective effect at 24 hours post-stroke does not necessarily translate into recovery of function in the subacute phase after stroke^24^. The dynamic development of injury extends the lesion size and spreads the injury to remote regions such as thalamus. All of these changes reflect a continuous impairment of neuronal function. Many pre-clinical studies have focused on the outcome during the acute phase post-stroke, but omitted the long-term outcome. Indeed, those pre-clinical strategies demonstrated reduced stroke volume during early phase, but failed in clinical translation^25^. Thus, in the present study, we investigated the effects of TRPV1-mediated hypothermia on functional recovery by a long-term serial assessment of behavioral function during post-stroke recovery. We demonstrated significant functional recovery in DHC treated stroke mice, suggesting that neuroprotection by a single treatment of TRPV1-mediated hypothermia is robust and lasting. Our findings validate the benefit of TRPV1-mediated hypothermia for treatment of ischemic stroke injury.

Focal cortical injury has been shown to trigger remote secondary damage in the thalamus following stroke or trauma^17,26,27^. Following primary injury to specific areas of the cortex, secondary thalamic neuronal loss first becomes evident after about three days and may progressively develop for several weeks^15–17^. The final neuronal damage in the thalamus may be the result of retrograde degeneration and anterograde Wallerian degeneration^28^. It should be noted that in our dMCAO model, thalamic injury is not a direct result of disrupted blood flow to the thalamus, as this region of the brain receives blood flow via small arteries arising from the posterior cerebral and posterior communicating arteries^27,29^. Instead, thalamic degeneration following cortical injury is thought to be conveyed via injury to long thalamic projections with terminations in the cortex (“thalamocortical projections”)^30^. These projections are not simply radial extensions from the thalamus (i.e. within the same coronal plane), but generally involve a progression to more anterior cortical regions – primarily S1/S2 sensory cortex and M1/M2 motor cortex^31^. We performed correlation analysis in Stroke/vehicle mice and found a significant positive correlation between thalamic injury and cortical injury from +1 mm plane sections, but not in the other three posterior sections. Note that these regions of injured cortex overlap with locations of previously described thalamic projections originating from the VPM and PoM thalamic nuclei^31^. Our findings reaffirm these thalamocortical projections between anterior cortex and thalamus in a pathological model.

In addition to the reduction of primary cortical injury, TRPV1-mediated hypothermia also reduced the development of remote thalamic injury – evident by decreased thalamic injury size, less neuron loss, and reduced astrogliosis. TRPV1-mediated hypothermia also reduced the thalamic fiber loss. The alleviation of both primary cortical injury and secondary thalamic injury may comprehensively contribute to the improved behavioral function that we observed. We further examined the relationship between cortical injury and thalamic injury in DHC-mediated hypothermia treated stroke mice. In contrast to the Stroke/vehicle group, DHC-treated mice demonstrated no correlation between cortical injury and thalamic injury. These findings suggest that the reduced thalamic injury with DHC-mediated hypothermia may disrupt progression of secondary injury downstream of primary injury.

Based on the pattern and location of dead neurons in the thalamus, we believe that the secondary injury involves retrograde injury to thalamocortical neurons which originate in the PoM and VPM nuclei and extend projections into the S1/S2 brain region. Cell death appears consistent with an apoptosis mechanism, as Nissl staining shows shrunken nuclei and nuclear condensation. The mechanism of how cell bodies in the thalamus become injured and how hypothermia attenuates injury in this region is currently undetermined. We presume that initial injury results from primary ischemic injury to axonal projections in the cortex (versus injury to corticothalamic neurons with projections into the thalamus) with subsequent degeneration of the thalamic cell bodies. However, it is not clear if a portion of these neurons with cortical projections survive with hypothermia treatment or if, alternatively, all neurons with cortical projections die, but with reduced injury to neighboring neurons in the thalamus (i.e. less collateral damage). Future studies are needed to define molecular events of this secondary injury and determine the effects of TRPV1-mediated hypothermia on its progression.

In summary, our study demonstrates that TRPV1-mediated hypothermia initiated 3.5 hours after cerebral ischemia significantly reduced both cortical injury (primary injury site) and thalamic injury (remote secondary injury) assessed at one month post-stroke. Of further note, we observed improved functional recovery with a single administration of TRPV1-mediated hypothermia. These findings provide further rationale for pursuing TRPV1 agonism as an adjunctive therapy for ischemic stroke.

## Methods

### Animals

All animal studies were approved by the Baylor College of Medicine Animal Care and Research Advisory Committee and in accord with National Institutes of Health Guidelines. Experiments were performed with male C57BL/6 mice, between 10–16 weeks and 21–32 g. All animals were housed in the animal care facility at BCM. Animals had access to food and water *ad libitum*. Animal rooms were maintained on a 12 hour light/dark cycle. Three groups were established: Sham (sham operated), Stroke/vehicle (cerebral I/R with vehicle), and Stroke/DHC (cerebral I/R with DHC). A total of 42 mice were used. A total of 4 mice in Stroke/DHC group did not fall within the targeted mild hypothermia temperature range (32–34 °C) and were therefore excluded from data analysis.

### Focal cerebral Ischemia/Reperfusion (I/R) mouse model

Focal cerebral I/R (dMCAO) was induced by transient occlusion of the left distal middle cerebral artery (MCA) and left common carotid artery (CCA) for two hours followed by reperfusion^9^. In brief, the distal MCA was accessed via a craniotomy and mechanically occluded just proximal to the anterior and posterior branches. Unlike the intraluminal filament model, this model results in an injury that is acutely restricted to the cortex^17^. Mice were anesthetized with isoflurane (5% induction and 2% maintenance in 100% oxygen) during the surgery. Thirty minutes before the end of the anesthesia, a dose of Buprenorphine-Opiod (0.1 mg/kg) was injected subcutaneously for pain relief during recovery. Following surgery, the mice were awakened and maintained normothermic (36 ± 0.5 °C body temperature) in a custom-built warming cage for the initial recovery period. Sham and Stroke/vehicle groups received heat support for the first four hours of reperfusion (or sham procedure), whereas the Stroke/DHC group received heat support only for the first 90 minutes of reperfusion so as to not interfere with the development of hypothermia (see below).

### Pharmacological hypothermia mouse model

Hypothermia was induced by continuous infusion of a TRPV1 channel agonist, dihydrocapsaicin (DHC, 1.25 mg/kg/hr, subcutaneously; Cayman Chemical Company, Ann Arbor, MI USA) for 8 hours by an osmotic pump (Alzet 2001D, Cupertino, CA USA) as previously described^9^. Vehicle consisted of 20% DMSO in saline. The infusion of DHC was initiated at the beginning of reperfusion, and preliminary experiments confirmed that the target mild hypothermia (32–34 °C) was reached at 90 minutes into the reperfusion period (3.5 hours after stroke onset). During the first four hours of the reperfusion in Stroke/DHC group, core body temperature was monitored every 15 min by implanted temperature transponder (Implantable Programmable Temperature Transponder; IPTT-300, BioMedic Data Systems, Seaford, DE USA) to ensure successful drop in core temperature within mild hypothermia range. The transponder was positioned under the back skin at the left side even with the kidney during the stroke surgery procedure. The osmotic pump was removed at 24 hours, in accordance with manufacturer recommendations. We did not observe any gross abnormalities during the DHC infusion period.

### Behavioral testing

Foot fault tests were performed at the same time of day by an investigator blinded to treatments to assess the behavioral function. Mice were pre-trained for three consecutive days before sham/stroke surgery. Testing results were recorded for the day before surgery and post-surgery days 3, 7, 14, 21 and 28. During the test, mice were allowed to freely move for two minutes on top of an elevated square grid (25 cm × 25 cm) with 1 cm spacing^32^. Each trial was recorded by GoPro camera (GoPro Hero 3) and videos were analyzed for foot fault. A “foot fault” was counted when the mouse foot slipped through the space of an elevated wire grid^33^. Total steps (movements of all limbs), contralateral side foot faults and total foot faults were counted. Contralateral foot fault% = contralateral side foot faults/total steps × 100%. The ratio of right side foot faults/total foot faults was calculated to identify the involvement of left (ipsilateral) brain injury.

### Histology

At 30 days post-stroke, mice were sacrificed by isoflurane overdose and then transcardially perfused with 30 ml cold saline. Brains were then fixed in 4% paraformaldehyde and embedded by paraffin. Serial 5 µm brain sections at +1 mm, 0 mm, −1 mm and −2 mm from bregma were collected by microtome and stored at room temperature. The sections were then deparaffinized with histo-clear, then sequentially rinsed in 100%, 95%, 70% of ethanol and ddH_2_O. Sections were then stained by 0.125% cresyl violet (Nissl staining, Sigma-Aldrich, St. Louis, MO).

Full section images were taken with a Zeiss LSM 780 Confocal Microscope using the plan-apochromat 20×/0.8 objective lens (Carl Zeiss, Germany). A total of 400 (20 × 20) tiled images were stitched together to get a single full high resolution image. Bright field images were acquired using an attached AxioCam ICc5 camera.

The cortical necrosis area and thalamic injury area were analyzed in Adobe Photoshop CS3. The cortical necrosis area at each serial position was quantified as [(contralateral cerebral cortex area - ipsilateral cerebral cortex area + infarcted cortex area)/contralateral cerebral cortex area] in percentage as described previously^34^. Supplemental Fig. 1 indicates these described areas on a representative Nissl-stained brain section. Infarct area was defined as the area of lost Nissl staining or area filled with dark pyknotic stained debris. On the sections taken at −2 mm from bregma, thalamic degenerative neuron injury area in the ipsilateral side was defined as abnormal neurons with darkly stained, shrunken nuclei, and atrophic perikaya^17^. The thalamic injury was calculated as [ipsilateral thalamic degenerative neuron injury area/contralateral hemisphere area] in percentage.

To evaluate a possible effect of DHC-induced hypothermia treatment on secondary injury, the correlation of cortical injury and thalamic injury were analyzed. Firstly, the relationship between cortical injury and thalamic injury were analyzed in Stroke/vehicle group mice to determine which region of the cortex is primarily responsible for secondary injury in the dMCAO injury model. The thalamic infarct (%) from the −2 mm section versus cortical infarct (%) from each of the coronal sections were plotted. Linear regression analysis was performed to determine which coronal section(s) demonstrated a clear correlation between cortical injury and thalamic injury (see Supplemental Fig. 2). The DHC-induced hypothermia treated brains for a similar correlation were then compared to deduce whether thalamic injury was likewise direcly correlated with the size of the primary cortical injury, or instead demonstrated evidence of downstream secondary protection (i.e. independent of cortical injury).

### Immunofluorescence

Brain sections of −2 mm from bregma were immunostained with antibodies for detection of neuron loss and astrocyte activation in the thalamus area. In brief, the 5 um thick paraffin sections were deparaffinized with histo-clear, then sequentially rinsed in 100%, 95%, 70% of ethanol and ddH_2_O. Epitope retrieval was performed by immersing slides in sodium citrate buffer (95°–100 °C) for 10 min. Sections were then incubated with 0.1% Triton X-100 in PBS for 10 min and blocked with 1.5% goat serum for 2 hours at room temperature. Sections were then incubated with primary antibody anti-GFAP (1:5000, Abcam, ab7260, Cambridge, MA) at 4 °C overnight and goat anti-rabbit IgG Alexa Fluor 594 (1:500, A11012, Invitrogen, Eugene, OR) for one hour at room temperature. The sections were incubated with pre-conjugated anti-NeuN Antibody (1:200, clone A60, Alexa Fluor 488 conjugated, Millipore, Billerica, MA) for 4 hours at room temperature. Sections were then mounted with Vectashield hardset mounting medium with DAPI (H-1500, Vector Laboratories, Burlingame, CA).

Immunolabeled brain slices were imaged with a Zeiss LSM 780 confocal microscope (Zeiss, Germany). A total of 5 Z planes with 7 × 7 tile images were stitched together. Representative images were presented by maximum projections using Zen silver edition software. A 500 × 500 µm^2^ square area was collected respectively at the posteromedial complex (PoM) and the ventral posteromedial (VPM) area from both ipsilateral and contralateral sides. Cells staining positive for NeuN, GFAP and DAPI were counted using ImageJ software (NIH).

### Diffusion Tensor Imaging (DTI)

At 30 days post-stroke, brain samples from a parallel cohort of mice were collected and prepared for DTI scanning as described previously^35^. Briefly, mice were transcardially perfused with 15 ml heparinized phosphate buffered saline (PBS, 20 unit/ml) followed by 15 ml 4% paraformaldehyde (PFA) for fixation. The mouse skull was exposed by removal of the skin, muscle, ears, nose tip, and lower jaw and fixed by 4% PFA at 4 °C overnight. After that, the head was incubated in 0.01% sodium azide (NaN_3_) in PBS at 4 °C for 7 days and then in 5 mM gadopentetate dimeglumine solution (Bayer HealthCare Pharmaceuticals Inc., Wayne, NJ) at 4 °C for 14 days. The head was equilibrated at room temperature for 6 hours before imaging.

All DTI scans were acquired on a 9.4 T Bruker Avance Biospec Spectrometer, 21-cm bore horizontal scanner with a 35 mm volume resonator (Bruker BioSpin, Billerica, MA) with Paravision 5.1 software (Bruker Biospin, Billerica, MA). The 3D DTI scan parameters were as follows: Spin echo, b-value = 0 and 1000 s/mm^2^, 20 diffusion directions with one non-diffusion weighted image, TR = 500 ms, TE = 14.8 ms, FOV = 1.5 × 1.0 × 2.0 cm, matrix = 164 × 96 × 96, NEX = 1, δ = 3 ms, Δ = 7 ms.

The image volumes were processed using DTI studio^36^ and ROIeditor (www.mristudio.org). The alignment was carried out using the DiffeoMap software^37,38^. The alignments were then applied to the tensor and the various maps (FA, colormap, B0, radial diffusivity), to yield aligned brain volumes. The inverse alignment transformation was applied to the template segmentation map, and then down-sampled to match the original subject resolution. This generated segmentation maps for the subject brains in their original scanned orientation and resolution. Fiber tracking was carried out using DTI Studio to quantitate fiber number and fiber length through specific brain regions (*e.g*. the thalamus). Amira (version 5.6; FEI) was utilized for presentation-quality visualization of FA maps and fiber tracking data.

### Statistical Analysis

Histology necrosis data was expressed as mean ± SEM. A t test was used for comparison between Stroke/vehicle and Stroke/DHC groups at each position. Pearson correlation analysis was used to evaluate the correlation between thalamic injury and cortical necrosis injury at each position from bregma in Stroke/vehicle group. Parallel line analysis was used to determine if the correlation of thalamic injury and cortical injury were significantly different in Stroke/vehicle and Stroke/DHC groups. Cell counting data was expressed as median ± SEM. A Kruskal–Wallis one-way ANOVA test was used to evaluate differences among three groups and a Mann–Whitney *U* test with Bonferroni correction was used for post hoc multiple comparisons. A t-test was used to compare fiber tract number between groups. All statistical calculations were performed using SigmaPlot 12.5 (Systat software). Behavioral data was expressed as mean ± standard error of the mean (SEM). Two-way repeated measure ANOVA was used for comparing groups with repeated measures. The Holm-Sidak method was used for all pairwise multiple comparisons.

### Data availability

All data generated or analyzed during this study are included in this published article and its Supplementary Data files.

## Electronic supplementary material

Supplementary Figures 1&2
