# Supplementary material for: TRPV1-mediated Pharmacological Hypothermia Promotes Improved Functional Recovery Following Ischemic Stroke
Source: Sci Rep. 2017 Dec 15;7:17685. doi: 10.1038/s41598-017-17548-y (PMC5732157; doi:10.1038/s41598-017-17548-y)

**TRPV1-mediated Pharmacological Hypothermia Promotes Improved Functional Recovery Following Ischemic Stroke**

Zhijuan Cao, Adithya Balasubramanian, Steen E. Pedersen, Jonathan Romero, Robia G. Pautler, and Sean P. Marrelli

**Supplemental Figure Legends**

Supplemental Fig 1 **Representative figure demonstrating the method of the cortical injury calculation**

(**A**) Representative image of Nissl-stained brain section at -2mm from bregma indicating the areas used for cortical injury calculation. The full section image was obtained by stitching together 400 tiled images (20x magnification). The outline in red indicates the contralateral cerebral cortex area; the outline in black indicates the ipsilateral cerebral cortex area; and the outline in green indicates the infarct area. Scale bar=1mm. (**B-E**) The enlarged images from figure A, present the details of the outlined area. Scale bar=200 µm.

Supplemental Fig 2 **No correlation between the secondary thalamic injury and the primary cortical infarct injury at posterior positions at one month post-stroke**

Thalamic injury area from -2mm section plotted versus cortical infarct area from (**A**) 0mm from bregma section); (**B**) -1mm from bregma; (**C**) -2mm from bregma in Stroke/vehicle group. (Pearson correlation analysis, R and P values are indicated within each plot, n=9, data are expressed as mean±SEM).

Supplemental Figure 1


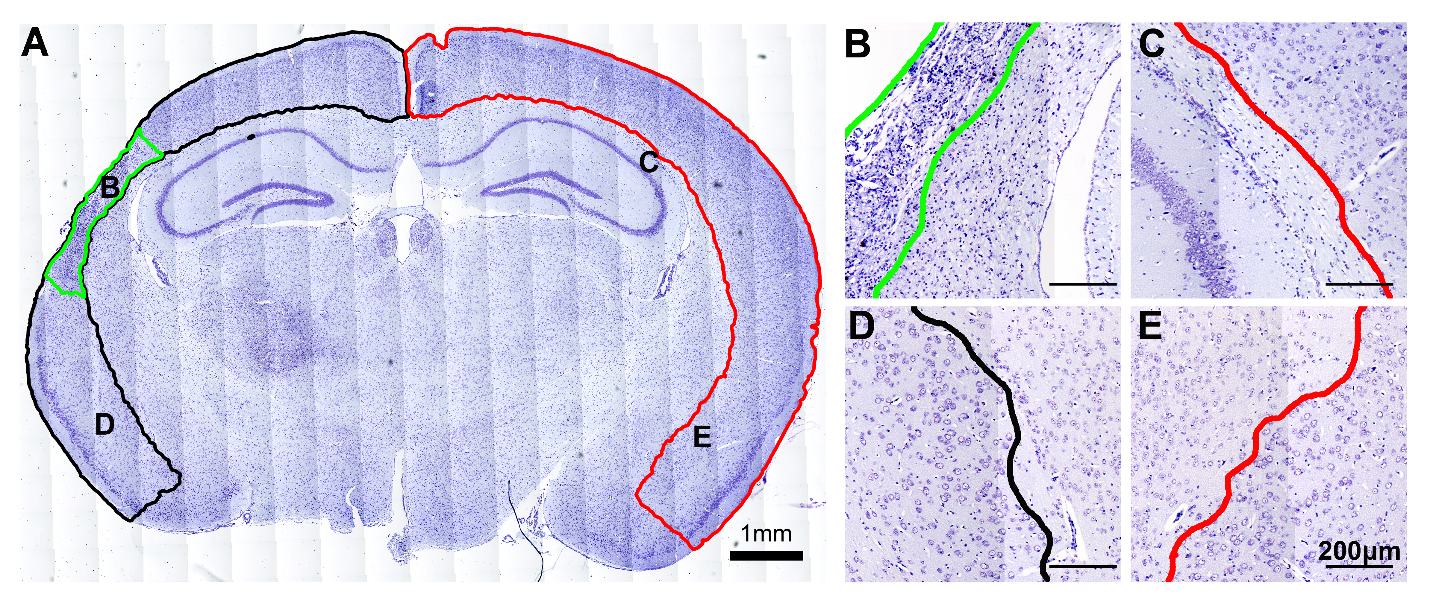


Supplemental Figure 2


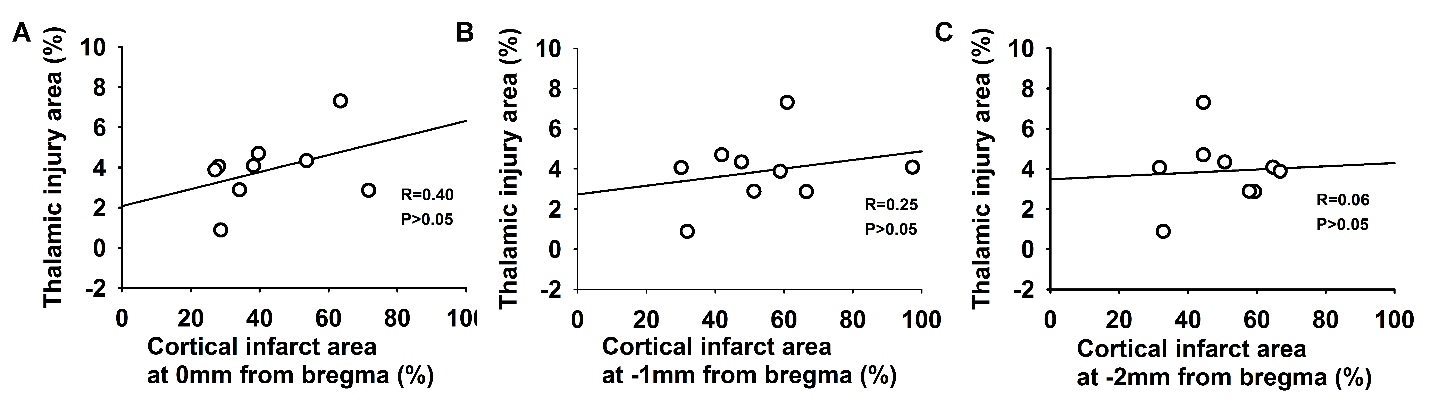

Supplement: Supplementary file 1 — Supplementary Figures 1&2 [file 41598_2017_17548_MOESM1_ESM.doc]
